# Supplementary material for: Content-rich biological network constructed by mining PubMed abstracts
Source: BMC Bioinformatics. 2004 Oct 8;5:147. doi: 10.1186/1471-2105-5-147 (PMC528731; doi:10.1186/1471-2105-5-147)
Supplement: Additional File 5 — The original Chilibot query results of the term "long-term potentiation (LTP)" and 22 other terms, limiting the latest references analyzed to the years 1990, 1995, 2000, and 2004. [file 1471-2105-5-147-S5.bz2 › chilibotAdditionalFile5/ltp2000/html/NMDA_ACTIN.html]

 


 **NMDA** and **ACTIN** 
  
Found 44 abstracts in PubMed,  **30 abstracts were retrieved and analyzed**.  


---

 Search Google  |
 PDF files only 
|  EDU domain only 

---

**Interactive relationship** (e.g. stimulation, inhibition, etc)

- The  **NMDA**  induced loss of spine  **actin**  was time and concentration dependent and blocked by  **NMDA**  receptor antagonists.  Ref: 9822742 J Neurosci, 1998
- The effect of  **NMDA**  on local  **actin**  assembly was strongly attenuated by pretreatment with an  **actin**  stabilizing compound or by an antagonist of the calcium dependent protein phosphatase calcineurin.  Ref: 9822742 J Neurosci, 1998
- Depolymerization of F  **actin**  led to a 40% decrease in both the number of synaptic  **NMDA**  receptor NMDAR1 clusters and the number of AMPA receptor GluR1 labeled spines.  Ref: 9502803 J Neurosci, 1998
- Here we show that  **actin**  dynamics in spines are potently inhibited by activation of either AMPA or  **NMDA**  subtype glutamate receptors.  Ref: 10966619 Nat Neurosci, 2000
- **NMDA**  receptor activation evokes an initial rapid depolymerization of F  **actin** .  Ref: 9307140 J Neurophysiol, 1997
- **NMDA**  peak current rundown has been attributed to calcium induced depolymerization of the  **actin**  cytoskeleton.  Ref: 9930735 J Neurochem, 1999
- Inactivation of  **NMDA**  receptors during hypoxia occurs by a variety of mechanisms, including alterations in the phosphorylation state of ion channel subunits, calcium dependent second messenger activation, changes in calcium dependent polymerization depolymerization of  **actin**  to postsynaptic receptors and activation of other G protein coupled receptors.  Ref: 9510526 J Exp Biol, 1998
- The results indicate that both spontaneous rundown and PKC potentiation of currents from heterologously expressed zeta1 epsilon2  **NMDA**  receptors depend on dynamic  **actin**  polymerization depolymerization but do not involve changes in microtubules.  Ref: 10505612 Neurosci Lett, 1999
- alpha Actinin 2, which binds both  **actin**  and  **NMDA**  receptors, dissociated from the receptor clusters, but PSD 95 remained associated with both the synaptic and nonsynaptic receptor clusters, consistent with a proposed cross linking function.  Ref: 9502803 J Neurosci, 1998
- Protein kinase C potentiation of currents from mouse zeta1 epsilon2  **NMDA**  receptors expressed in Xenopus oocytes depends on f  **actin**  g  **actin**  cycling.  Ref: 10505612 Neurosci Lett, 1999
- **Actin**  filament F  **actin**  depolymerization leads to the use dependent rundown of N methyl D aspartate  **NMDA**  receptor activity in rat hippocampal neurones.  Ref: 10455249 Br J Pharmacol, 1999
- We suggest that the function of somatodendritic  **NMDA**  receptor channels in rat hippocampal neurones can be modulated by RhoA via its action on F  **actin** .  Ref: 10455249 Br J Pharmacol, 1999
- The averaged apparent intensity of soma associated F  **actin**  derived fluorescence F app was measured from fixed cells after incubation in either 1 normal Krebs solution containing 2 mM extracellular calcium calcium ex or 2 normal Krebs solution plus N methyl D aspartate  **NMDA**  for 2 min immediately before fixation.  Ref: 9307140 J Neurophysiol, 1997

**Parallel relationship** (e.g. studied together, co-existance, homology, etc.)

- Depolymerizing F  **actin**  did not affect  **NMDA**  evoked whole cell currents.  Ref: 10627577 J Neurosci, 2000
- The synaptic clustering of PSD 95, a putative  **NMDA**  receptor anchoring protein and a core component of the postsynaptic density PSD, was unaffected by  **actin**  depolymerization, microtubule depolymerization, or detergent extraction.  Ref: 10844024 J Neurosci, 2000
- Alpha actinin alpha actinin 2 is a protein which links the NR1 and NR2B subunits of N methyl D aspartate  **NMDA**  glutamate receptors to the  **actin**  cytoskeleton.  Ref: 10925145 Brain Res Mol Brain Res, 2000
- Shank may function as a scaffold protein in the PSD, potentially cross linking  **NMDA**  receptor PSD 95 complexes and coupling them to regulators of the  **actin**  cytoskeleton.  Ref: 10433268 Neuron, 1999
- Recently the  **actin**  binding protein alpha actinin 2 was shown to bind directly to the  **NMDA**  receptor subunits NR1 and NR2B, suggesting that alpha actinin 2 may function to attach  **NMDA**  receptors to the  **actin**  cytoskeleton.  Ref: 9454847 J Neurosci, 1998
- We have investigated, by means of the whole cell patch clamp technique, whether the  **actin**  fibres which interact with  **NMDA**  receptors are controlled by Rho GTPases.  Ref: 10455249 Br J Pharmacol, 1999
- Role of  **actin**  in anchoring postsynaptic receptors in cultured hippocampal neurons differential attachment of  **NMDA**  versus AMPA receptors.  Ref: 9502803 J Neurosci, 1998
- These results suggest that subcellular clustering of the NR1 subunit of the  **NMDA**  receptors is regulated by the interaction of its C terminus with cytoskeletal components, where differentially spliced cassettes interact separately with  **actin**  filaments or microtubules.  Ref: 10515258 Neurosci Res, 1999
- We used  **actin**  perturbing agents and detergent extraction of primary hippocampal cultures to test directly the role of the  **actin**  cytoskeleton in localizing GABAA receptors, AMPA and  **NMDA**  type glutamate receptors, and potential anchoring proteins at postsynaptic sites.  Ref: 9502803 J Neurosci, 1998
- It is shown that N methyl D aspartate  **NMDA**  receptor stimulation reversibly translocates green fluorescent protein tagged CaMKII from an F  **actin**  bound to a PSD bound state.  Ref: 10102820 Science, 1999
- Since the activity of the  **NMDA**  R channel is dependent on the integrity of  **actin**  and leads to calpain mediated spectrin breakdown, we have investigated whether the  **actin**  binding spectrin may interact directly with  **NMDA**  Rs.  Ref: 9670010 EMBO J, 1998
- However, if the F  **actin**  polymerization inhibitor cytochalasin D was added to cells immediately after  **NMDA**  was removed, the F app did not recover with time 36 3%.  Ref: 9307140 J Neurophysiol, 1997
- Therefore, our results indicate that although calbindin may not influence calcium dependent regulatory processes occurring very near the  **NMDA**  receptor channel, it appears to be more effective at buffering local elevations in intracellular calcium at the  **actin**  cytoskeleton.  Ref: 9930735 J Neurochem, 1999
